# Modifying Adhesive Materials to Improve the Longevity of Resinous Restorations

**DOI:** 10.3390/ijms20030723

**Published:** 2019-02-08

**Authors:** Wen Zhou, Shiyu Liu, Xuedong Zhou, Matthias Hannig, Stefan Rupf, Jin Feng, Xian Peng, Lei Cheng

**Affiliations:** 1 State Key Laboratory of Oral Diseases, Sichuan University, Chengdu 610041, China; zhouwendentist@139.com (W.Z.); liusy9307@163.com (S.L.); zhouxd@scu.edu.cn (X.Z.); jinjinfeng@sohu.com (J.F.); 2 Department of Cariology and Endodontics, West China School of Stomatology, Sichuan University, Chengdu 610041, China; 3 National Clinical Research Center for Oral Diseases, Sichuan University, Chengdu 610041, China; 4 Clinic of Operative Dentistry, Periodontology and Preventive Dentistry, Saarland University Hospital, 66421 Homburg/Saar, Germany; Matthias.Hannig@uniklinikum-saarland.de (M.H.); Stefan.Rupf@uniklinikum-saarland.de (S.R.); 5 Department of Geriatric Dentistry, West China School of Stomatology, Sichuan University, Chengdu 610041, China

**Keywords:** resinous restorations, dental adhesives, MMPs inhibition, antibacterial, remineralization, longevity

## Abstract

Dental caries is a common disease on a global scale. Resin composites are the most popular materials to restore caries by bonding to tooth tissues via adhesives. However, multiple factors, such as microleakage and recurrent caries, impair the durability of resinous restorations. Various innovative methods have been applied to develop adhesives with particular functions to tackle these problems, such as incorporating matrix metalloproteinase inhibitors, antibacterial or remineralizing agents into bonding systems, as well as improving the mechanical/chemical properties of adhesives, even combining these methods. This review will sum up the latest achievements in this field.

## 1. Introduction

Dental caries is prevalent all around the world, and resin composites are widely used as filling materials [1,2]. However, longevity of resinous restorations is not satisfactory. The failure rate ranges from 15% to 50% according to previous survival investigations [3,4]. The failure mainly due to secondary caries, fracture, marginal deficiencies, wear, and postoperative sensitivity [5,6,7].

In addition, resin-dentin bonds are less durable than resin-enamel bonds, because heterogeneity of the structure and composition of dentin [8]. The failure of resin-dentin bonding will result in microleakage, staining, recurrent caries, and postoperative sensitivity [9], and the interaction of these situations can accelerate the degradation and failure of resin-dentin bonding.

**Figure 1:**
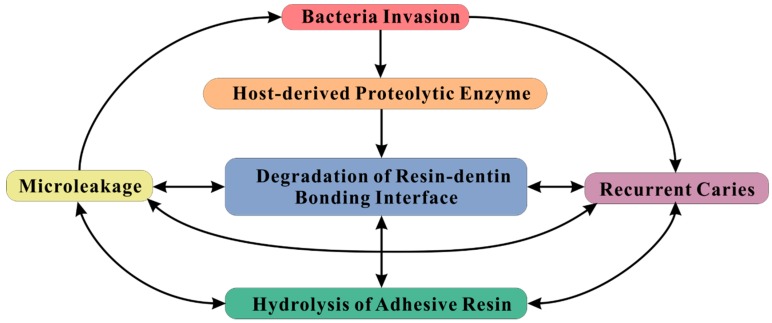
Factors involved in failure of resin-dentin bonding. The arrows represent aggravation effects of the respective factors.

Therefore, it is necessary to refine the adhesive materials for breaking this vicious circle (Figure 1) [10,11]. Numerous studies have been designed in pursuit of more stable resin-dentin bond. In this review, the modifying methods of adhesive materials to improve the longevity of resinous restorations are summarized.

## 2. The Development of Dentin Adhesives

The earliest pursuit of satisfactory resin-dentin bonding began in the early 1950s and has reached its eighth generations (Table 1) [12,13]. Current adhesives tend to be simplify bonding steps and make them more user-friendly [14].

Kramer and McLean were among the first to use glycerophosphoric acid dimethacrylate (GPDM) to bond to dentin [15]. Then, Buonocore made attempts at etching dentin with 7% hydrochloric acid and apply GPDM bonding resin in 1955 [1,16]. His work laid the foundation for adhesive restorative and preventive dentistry [13].

The second generation of dentin bonding systems was developed in the early 1980s. These bonding adhesives were mainly composed of halophosphorous esters of unfilled resins, such as bisphenol-glycidylmethacrylate (BisGMA) and hydroxyethyl methacrylate (HEMA) [13]. The bonding depended on a surface wetting phenomenon and ionic interaction between the phosphate groups and dentinal calcium [13,17].

Preparing dental cavities form a layer of cutting debris on instrumented dental surfaces which is termed smear layer [9]. The first and second-generation dentin bonding agents chemically bonded to the smear layer rather than to the dentin tissue. Therefore, the bond strength formed in this way was too weak to resist the polymerization shrinkage and hydrolysis of composite resin, leading to the poor clinical performance of these adhesives [13,18,19].

In order to remove and/or modify the smear layer the third-generation adhesives were designed in the late 1980s [13,19]. With these systems, the acid etching of the dentin partially opening dentinal tubules increasing the permeability of resins and adhesives. These third-generation adhesion systems mostly use a hydrophilic dentin-resin primer that can infiltrate and soften the smear layer, modifying it and promoting adhesion to dentin. Moreover, the hydrophilic group such as hydroxyethyl trimellitate anhydride (4-META) and biphenyl dimethacrylate (BPDM) create chemical adhesion to the unfilled adhesive resin [19].

Even though these adhesives do not completely eliminate marginal leakage, they are more effective than their predecessors [13].

Following these pioneer approaches, numerous studies were devoted to improving dentin bonding capabilities and great advances have been achieved [1]. The next generation of dentin adhesives introduced the concept of total-etch technique, substantially improving retention of adhesive restorations to the enamel and dentin [13,19,20].

The total-etch technique permits the etching of enamel and dentin simultaneously with phosphoric acid. The mineralized tissues of the peritubular and intertubular dentin are dissolved by the acidic action with the smear layer being completely removed. The primer of this generation consists of a solvent with one or more hydrophilic resin monomers which contain two functional groups—a hydrophilic group and a hydrophobic group. The hydrophilic group has an affinity for the dentin surface and the hydrophobic (methacrylate) group has an affinity for resin. Consequently, the primer increases the surface energy and wettability of the dentin surface [13]. The unfilled resin bond agents diffuse into the exposed collagen fibril scaffold, hybridizing or micro-mechanically interlocking with these tooth tissues. Thus, a layer of collagen and resin commonly called the “hybrid layer” was formed. Nakabayashi et al. were the first to report this concept in 1982. Nowadays the hybrid layer is widely accepted and thought to be the primary bonding mechanism of resin-dentin bonding [13,19].

These multi-step dental adhesives are considered as the ‘gold standard’ adhesives. Many studies have shown that these materials provide better marginal seal and shear bond strengths [13]. However, the multi-step clinical application of these systems is complex and time consuming [18].

For more user-friendliness and less time effort, the total-etch technique was simplified into two steps by combining primer and bonding resin into one application [9,18,20,21,22]. Modifications of these adhesives are designed as fifth-generation dentin bonding systems [22]. Although these adhesives often been called single-bottle systems, they require a separate enamel and dentin conditioning step, and some require multiple applications of the adhesive [13,18]. The application is sensitive with regard to technique, resulting in variation of bond strengths achieved by different clinicians [13].

In order to achieve a proper bond to dentin using only one bottle [19], self-etch adhesives were introduced into the market. These can etch and prime simultaneously and dissolve the smear layer with acidic monomers, thus enabling a single application procedure of a so-called ‘all-in-one’ adhesive [9]. Self-etch adhesives can be either one- or two-step systems depending on whether the self-etching/primer solution is separated from the bonding agent or combined with it [9,20,21]. Self-etch adhesives demineralize and infiltrate into the dentin surface simultaneously to the same depth, theoretically preventing incomplete penetration of the adhesive into the exposed collagen network. These systems contain specific functional monomers such as 10-MDP, 4-MET, and phenyl-P with carboxylic and phosphate groups. These functional groups are able to ionically bond with calcium in hydroxyapatite providing satisfactory chemical bonding to dentin [9].

Even though adhesive systems have been significantly improved, the bonded interface remains the weakest area of resinous restorations [1]. Large number of studies confirm that three-step total-etch adhesives still have the best durability [9], followed by two-step self-etch adhesives, and the one-step adhesives is the least durable [1,14,23,24]. The hydrophilic nature of self-etch adhesive systems is considered to be responsible for the poor clinical performance, these systems are more prone to water sorption, hydrolytic breakdown and loss of the structural integrity at the resin-dentin bonding interface [9,21]. Nevertheless, the self-etch approach may have the best future perspective due to its reduction of application time and its favorably low technique-sensitivity [24].

In 2010, voco America introduced voco futurabond DC as eighth-generation bonding agent, which may be used either as etch-and-rinse or as self-etch adhesives with the same single bottle of adhesive solution [12,25]. Monomers that are capable of producing chemical and micromechanical bond adhesion to the dental substrates were incorporated. Such as methacryloyloxydecyl dihydrogen phosphate (MDP), which can bond ionically to calcium found in hydroxyapatite [12,26]. In addition, the matrix of universal adhesive contains monomers of hydrophilic (hydroxyethyl methacrylate, HEMA), hydrophobic (decandiol dimethacrylite, DDMA) and intermediate (bis-GMA) nature. This combination enables the formation of bridge over the gap between the hydrophilic tooth substrate and hydrophobic resin restorative [12]. Studies indicated that universal adhesives introduce versatility without compromising its bonding effectiveness [25,27,28]. Because universal adhesives are marketed in short order, in vivo and in vitro long term studies are required to evaluate its effect in establishing a long-term success of composite restoration [12].

With the long time development of dentin adhesives, it has been widely accepted that the resin-dentin bonding is mainly determined by three aspects: infiltration of monomers into the demineralized intertubular matrix, formation of hybridized intratubular resin tags, and chemical bonding to the dentin surface [29].

## 3. The Factors Related with the Failure of Resin-Dentin Bonding

### 3.1. Degradation of Resin-Dentin Bonding Interface

#### 3.1.1. Host-Derived Proteolytic Enzymes

Demineralized dentin collagen matrix acts as a scaffold for resin infiltration during the resin–dentin bonding procedure, forming the hybrid layer which is paramount to dentin bonding strength [30]. Therefore, degradation of collagen matrices by matrix metalloproteinases (MMPs) and cysteine cathepsins are believed to be among the major reasons for the failure of resin restorations [1,31,32,33,34].

MMPs are synthesized by odontoblasts and trapped within mineralized dentin matrix. These proteolytic enzymes are calcium- and zinc-dependent [34]. MMPs and cysteine cathepsins can attack type I collagen, the most abundant organic component of dentin matrix [34], in both helical N- and C-terminal portions [31]. They can be activated by proteinases, chemical agents, low pH, heat treatment, as well as mechanical stress [34,35]. Acid-etchants used in dentin bonding and weak acids released by cariogenic bacteria can uncover and activate matrix-bound MMPs. Incomplete resin infiltration also contributes to their activation [34,36,37]. The exposed dentin collagen loses its protective triple helical conformation and presents the recognizable and available cleavage sites, becoming more vulnerable to MMPs and cathepsins [38].

#### 3.1.2. Adverse Chemical/Biochemical Interactions

It has been demonstrated that hydrolysis of hydrophilic resins could compromise the durability of resin-dentin bonding [32,37,39,40,41]. First of all, pulpal pressure, equivalent to approximately 15 cm H_2_O may drive the dentin fluid through dentinal tubules to the surface of the bond interface, promoting the degree of hydrolysis of resin tags and reducing the sealing ability of adhesives [39].

This is aggravated by methacrylate adhesives containing a number of ester bonds that are subject to chemical and/or enzymatic hydrolysis [42]. Human saliva contains numerous cholesterol esterase and pseudocholinesterase, which act synergistically to degrade dimethacrylates [41]. This fact was demonstrated by some in vitro studies in which the resin-dentin bond was aged with artificial saliva containing cholesterol esterase, which greatly decreased the microtensile bond strength [23].

It is well known that dentin is hydrophilic [39], so it is difficult to combine a hydrophobic adhesive resin with dentin [43]. Furthermore, the hydrophilic polymers promote water sorption that accelerates the speed by which the polymers plasticize, lowering their mechanical properties [44].

The presence of residual monomer can have a plasticizing effect on the polymer [45] and toxicity against pulp cells [46], thereby a low degree of conversion is one of the major factors lead to the failure of dental adhesives [47].

#### 3.1.3. Mechanical Loading

The importance of mechanical loading in dentin has not been fully elucidated. It has been demonstrated that masticatory forces can adversely affect the bonding interface, bending the tooth structures, leading to gap formation and marginal leakage around restorations [22,48].

It has been speculated that mechanical loading or some other stimulation may activate the dentin collagen matrix anchored bioactive molecules and signaling factors, which might be involved in mineralization, tissue regeneration, cells differentiation and other biological functions [49].

Wood et al. speculated that exposed collagen fibrils, due to incomplete infiltration of bonding resin, are vulnerable to creep or cycling fatigue during function. Additionally, surface strains of the adhesive layer would cause Poisson’s effects, facilitating the absorption of fluids and accelerating degradation of the adhesive [31].

### 3.2. Microleakage

When polymerization shrinkage and the associated contraction stresses of composite resins are higher than the bond strength, marginal gaps would form at the interface [50,51], resulting in microleakage [8], recurrent caries [52,53], and pulpal inflammation [20,21].

Bacterial enzymes and metabolite, bacteria and oral fluids can penetrate into the tooth-composite crevices [21,51,54,55]. Bacterial enzymes may be involved in the degradation of the hybrid layer [40]. Transmission electron microscopy images have indicated that collagenases from bacteria have the ability to increase nanoleakage at the dentin bonded interface [23]. Furthermore, weak acids, such as lactic acid produced by cariogenic bacteria, can activate MMPs, impairing resin-dentin bond durability [37]. The negative effect of cholesterol esterase in saliva has been discussed earlier in this article.

In addition, cavity disinfectants could not completely eliminate the viable microorganisms in the prepared tooth cavity and offer long-term antibacterial effects, the residual bacteria in the tooth cavity can magnify the problems associated with microleakage [20,21] and lead to caries and pulp inflammation [20,52,53,56].

The breakdown of dentin bonded interface sealing poses a challenge to the longevity of restorations [8]. Improvements in bonding agents might give rise to better marginal sealing and less margin failure [53].

### 3.3. Dental Pulp Response to the Adhesive System

One reason for failure of the restorative intervention of caries is pulpal problems occurring after treatment [14,51,57]. The elements which influence pulpal response to resinous restorations are as follows: first, the trauma of tooth tissue during cavity preparation; then, the toxic effects of the restorative materials; and the third one is the indirect inflammatory influence of microleakage [51].

Biocompatibility of dentin bonding agents can affect this treatment outcome. According to previous studies, components of dentin bonding agents such as acids, monomers and co-monomers have potential for pulpal toxicity [14,51,56]. Resin monomers, such as bisphenol-glycidylmethacrylate (BisGMA), triethylene glycol dimethacrylate (TEGDMA), and 2-hydroxyethyl methacrylate (HEMA) are the main toxic components in adhesive systems [58,59]. Their cytotoxic effects depend on the amount of non-polymerized monomers and dentin permeability and duration of perfusion [51,58,60,61]. They can impair regenerative and reparative capacities of the dentin-pulp complex [62].

Thus, the remaining dentin thickness also affects the intensity [63]. In addition, the smear layer produced during the cavity preparation procedure can reduce the dentin permeability, self-etch adhesives and self-adhesive cements would alter, but not remove, the smear layer [64]. In contrast, total-etching adhesives may eliminate the smear layer, this may explain the fact that these systems create more irritation to the pulp than self-etching adhesives system [60].

## 4. Methods to Modify the Bonding System

### 4.1. Adhesive Materials with Anti-Matrix Metalloproteinase Functions

**Figure 2:**
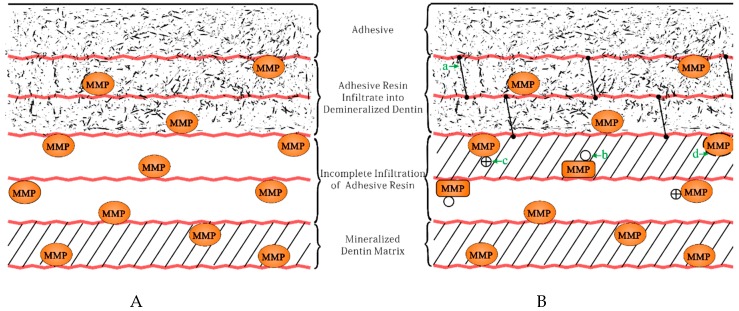
Schematic drawing presents the mechanism of MMPs-inhibiting adhesives. (**A**) The common condition of resin-dentin bonding; (**B**) The different way of inhibiting MMPs. (a) Cross-linking of collagen. (b) Changing conformation of MMPs. (c) Electrostatically binding to catalytic site of MMPs. (d) Entrapping MMPs and collagen in the newly-formed crystal.

Applying MMPs inhibitors as a component of the adhesive is an approach with great promise [32,65]. By this way, bond systems were provided with MMP-inhibitory functions which may help to improve durability of adhesive restorations [38]. Such a kind of adhesives has the potential to decrease the degradation of the collagen fibrils within the hybrid layer via inhibiting the host-derived collagenolytic activity [1] (Figure 2).

#### 4.1.1. Mechanism of Matrix Metalloproteinase Inhibition

One of the mechanisms of MMPs inhibition is cationic-anionic reaction, cationic agents like chlorhexidine (CHX) may electrostatically bind to negatively-charged catalytic sites of MMPs, blocking the active site [66,67]. Chelating or coordinate covalence bond with zinc or calcium present in the catalytic domain also leads to loss of catalytic activities of MMPs [35,68,69].

Another mechanism of MMPs inhibition is protein cross-linking, cross-linkers can induce conformational changes in MMPs 3D structure and hindering molecular mobility which is essential for their enzyme activity [41,58,70]. In addition, activity of MMPs can be interfered with altering circumstances around MMPs like pH and concentration of Zn^2+^ [71,72].

#### 4.1.2. Collagen Cross-Linking Agents

Protein cross-linking agents were proposed to induce conformational changes in MMPs 3D structure [41,70] and cause MMPs to lose molecular mobility [58]. At the same time, protein cross-linking agents may stabilize collagen matrix and improve the mechanical properties of the hybrid layer, thus strengthening the resin-dentin bond [32].

In previous studies, cross-linking agents have been incorporated into dentin bonds to improve the resin-dentin bonding. For instance, grape seed extract (GSE) is composed mainly of oligomeric proanthocyanidins (PA), which is a natural collagen cross-linker [32,73]. More importantly, PA has been shown to inhibit production and activity of MMPs [40].

Another cross-linking agent, hesperidin (HPN), hesperetin-7-O-rutinoside, is a flavonoid extract of citrus fruits [32,74]. Islam et al. incorporated HPN and GSE (0.5 wt%) into Clearfil SE primer (Kuraray Medical, Inc.), respectively. They found that HPN exerted a positive influence on the immediate resin-dentin bond strength, the hardness and elastic modulus of the interface, but GSE showed the opposite effect on the bond strength [32]. Nevertheless, the results of GSE are controversial [32,40,75]. The utilization of different adhesive systems may result in such different findings [32]. Adding GSE into model bonding agents with compositions similar to Single Bond Plus (3M ESPE Dental Products, St. Paul, MN, USA) at 5.0 wt% may increase collagen degradation resistance [40]. But as the fraction increased, the bonding strength would be weakened. This drawback may be due to the large molecular size of PA, which might have hindered the etching effect of self-etch primer [32,73]. As PA has capability to quench reactive oxidative radicals, increasing the content of PA (>10 wt%) will hamper the polymerization of adhesives [47].

#### 4.1.3. Alcohols

Tezvergil-Mutluay et al. found that alcohols can inhibit MMPs in vitro. Probably by forming a coordinate covalence bond between the MMP’s catalytic zinc and the oxygen atom of the alcohol’s hydroxyl group and the bonding might be attributed to the hydrophobic nature of alcohols [66].

As is known, HEMA, an ethanol-ester of methacrylic acid, is a common ingredient of bonding agents. Dissolving HEMA into primers with polar solvents in a proper fraction may help forming a stronger hybrid layer, thereby improving the bond strength and reducing stress transfer [76,77]. However, once HEMA copolymerized with other adhesive monomers, it loses its MMP inhibition ability.

### 4.2. Adhesives with Remineralization Functions

Even though protease inhibitors or cross-linkers can block degradation of collagen matrices, they cannot restore the demineralized hybrid layer [78]. Furthermore, cariogenic bacteria produce acids, causing demineralization of the tooth structure and the tooth-restoration margins [79]. A new approach to stabilize resin-dentin bonding is to remineralize the hybrid layer [78].

#### 4.2.1. Mechanism of Adhesives with Remineralization Functions

The adhesive systems with capacity of remineralization are believed to increase the life expectancy of adhesive restorations via autonomously healing the microcracks and neutralizing acidic acids within the adhesive joint [14]. A low pH could cause demineralization, it shifts the equilibrium dissolution reaction of hydroxyapatite (HA) towards demineralization [80]. Therefore, to combat demineralization, it would be beneficial to raise the pH. The adhesives with remineralization functions could providing alkaline ions like Ca and P to neutralized the acid [81,82]. These materials could help to the epitaxial growth of the remaining HA crystals in the partial demineralized dentin regions. Particularly they could serve as nuclei in excess demineralized dentin regions lacking the HA crystal nuclei. Thus, demineralized zones in hybrid layer region could be remineralized [83].

#### 4.2.2. Modify Adhesives with Remineralization Functions

In the study of Hashimoto et al., commercially available fluoride-releasing resin adhesives (OptiBond Solo and Reactmer Bond) showed self-reparative ability with regard to bond leakage, by inducing crystal growth within gaps of resin-dentin interfaces [84].

Bioactive glass (BAG), calcium phosphate (CaP), and HA could potentially be used as a source of CaP and as an adjunct to biomimetic remineralization, allow epitaxial deposition of CaP on their external surfaces [83]. The endogenous MMPs fossilized by the growth of apatite mineral, consequently protecting collagen fibrils [85].

Apart from remineralization, BAG has anti-microbial activity, due to increasing the pH in their environment via the continuous release of alkaline elements: Na^+^ or K^+^ exchanged with H^+^ or H_3_O^+^ ions [72,86]. It was also hypothesized as the possible mechanisms of MMP inhibition, since optimum MMP activity occurs at pH 7, that activity would be greatly slowed at alkaline condition [72]. The bioactivity of BAG could be potentiated by doping with specific functional and therapeutic ions, such as zinc, silver, various silica, niobium, fluoride, and copper (Cu), etc. [80,85,87]. For example, incorporation of 30 wt% niobium-based bioactive glass exerts radiopacity to the adhesive system without influencing bioactive properties, microhardness, bond strength, and degree of conversion [85]. In addition, Cu^2+^ is considered a potent inhibitor of MMPs in human dentin, Jun et al. demonstrated Cu^2+^ releasing nano-bioactive glass added adhesive showed MMP deactivation and remineralization properties at the resin-dentin interface [88].

CaP are the main constituents of the mineral phase in bones and teeth, and have been extensively investigated in remineralization of dentin [89]. Adding α-tricalcium phosphate (α-TCP) nanofiller into adhesive resins can improve bond strengths [90]. Furthermore, nanoparticles of amorphous calcium phosphate (NACP) could be incorporated into the adhesive up to 40 wt% without affecting bond strength [52,81]. NACP modified adhesives could greatly increase the Ca and P ion release with NACP filler level and at cariogenic low pH [79,91]. Ca and P would re-precipitate to form hydroxyapatite, significantly increasing the mineral content of the lesion [92].

Hypotheses have been proposed that HA nanorods in adhesives may biomineralize with the collagen network of tooth through hydrogen bonding between COOH, OH, and NH_2_ of the collagen network and OH group of the hydroxyapatite particles, achieving remieralization of the dentin at resin-dentin interface [93]. It was demonstrated that adding 7 wt% nano-sized HA fillers to SBMP (3M, St Paul, MN, USA) adhesive resin contributed positively to the immediate micro-tensile bond strength values in the dentin [83].

Another advantage of adhesives with remineralization functions is that they can entrap MMPs and collagen in the newly formed crystal, accordingly fossilizing and inhibiting the MMPs and cathepsins in the demineralized dentin tissues or lesions and protecting the collagen from degradation [41].

### 4.3. Antibacterial Bond System

The agents used to modify adhesives have different antibacterial mechanism. An antibacterial primer would help to kill residual bacteria in the tooth cavity [94], and antibacterial bonding agents could combat biofilms and recurrent caries at the tooth-composite margins [94,95].

Ag ions could interact and inactivate the vital enzymes of bacteria, and cause the DNA in the bacteria to lose its replication ability, leading to cell death [55,56,79].

The nanoparticles of silver (NAg) had a high surface area, a low filler level of it in the adhesives could provide potent antibacterial effects, without affecting the color and mechanical properties of dentin adhesives [56,94].

Chitosan is a collective name to describe a family of linear polysaccharides composed of β-1→4 linked d-glucosamine, with some residual interspersed N-acetyl-d-glucosamine [43]. It has been introduced into dental bonding systems due to its antibacterial properties. But the bonding strength significantly decreased with increasing the concentration of chitosan [67]. To deal with this drawback, Diolosà et al. synthesized a primer based on methacrylate-modified chitosan, which possessed both hydrophilic and hydrophobic features, and could interact with both the restorative material and the organic part of the demineralized tooth [43].

### 4.4. Multiple Effective Adhesives

The reasons for unsatisfactory resin-dentin bond durability are complex. Adhesives with just one particular property are not able to solve this issue effectively. Thus, multiple function bond systems are increasingly desired.

#### 4.4.1. Quaternary Ammonium Salts

Quaternary ammonium salts (QAS) are widely used in water treatment, surface coatings, and the food industry due to their low toxicity and potent antimicrobial activity [15].

QAS are positively charged, when negatively-charged bacteria cell contact the positive quaternary amine charge (N^+^), the alteration of membrane permeability or surface electrostatic balance of bacteria will take place, leading to cytoplasmic leakage under its own osmotic pressure [54,56,79,94,96]. This is believed to be the antibacterial mechanism of QAS.

There is another hypothesis about antibacterial effect of QAS. The positively charged QAS in dentin adhesives may facilitate negatively charged bacterial initial adherence [97]. However, the synthesis of extracellular matrix, which is believed to provide adherent sites for growth and proliferation accumulation of bacteria, might be inhibited. Moreover, positively-charged QAS can interact with the negatively charged EPS, changing the physicochemical properties of EPS so as to reduce bacterial adherence [97,98].

In addition to the ability to kill bacteria, the cationic quaternary ammonium methacrylates (QAMs) may electrostatically bind to negatively charged catalytic sites of MMPs, which contain cysteine-rich repeats, blocking the active site [99]. QAMs also have been proven to inhibit the activity of cathepsin [31,99]. Li et al. demonstrated that 12-methacryloyloxydodecy l-pyridiniumbromide (MDPB) [31] and a new QAMs monomer dimethylaminododecyl methacrylate (DMADDM) have strong inhibitory effects on MMPs [65].

**Figure 3:**
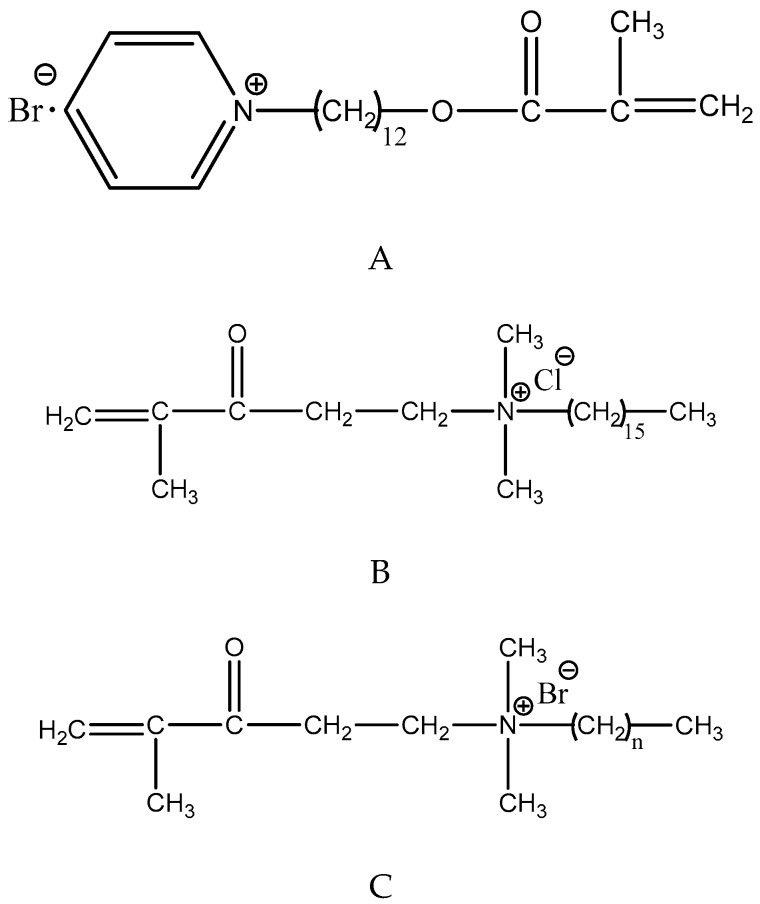

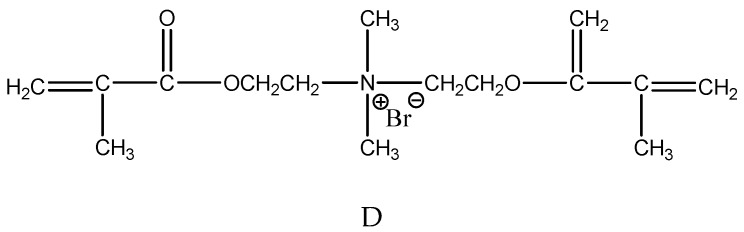
Chemical structures of different quaternary ammonium monomers. (**A**) Methacryloyloxy-dodecylpyridinium bromide (MDPB); (**B**) methacryloxylethylcetyl ammonium chloride (DMAE-CB); (**C**) *n* = 11, dimethylaminododecyl methacrylate (DMADDM), *n* = 15, dimethylaminohexadecyl methacrylate (DMAHDM); and (**D**) quaternary ammonium dimethacrylate (QADM).

QAMs were incorporated into adhesive agents for their antibacterial functions [15,54,95,96] (Figure 3). It has been demonstrated that bonding systems containing QAMs showed a significant antibiofilm activity [54]. The antibacterial monomers based on quaternary ammonium immobilized by polymerization in bonding agents would not leach from the hybrid layer [20]. Thus, the modified liquid could act as a primer, a cavity disinfectant as well as long-term antibacterial agent [55,56,94]. Imazato et al. first incorporated a quaternary ammonium monomer, MDPB, into the bonding system. They used LB bond (Kuraray, Osaka, Japan) as the parental agent: the primer was modified with MDPB at 5 wt% and 2.5 wt% for the bonding resin. They performed a series of in vitro and in vivo studies to investigate the MDPB-containing LB dentin adhesives [100,101,102].

The minimum bactericidal concentration values of MDPB against seven oral streptococci were reported within 31.3–62.5 mg/mL [98]. Before polymerization, MDPB in primer acts as cavity disinfection [20], after curing the primer still has the antibacterial properties [1,100,103]. Additionally, the cured adhesive resin with MDPB also exhibited a bacteriostatic effect on *S. mutans* [100]. With antibacterial activity before and after curing, the application of an MDPB-containing adhesive is promising to manage secondary caries, even root surface caries and active lesions [20].

The results from tensile bond strength measurement of the MDPB-containing bonding system indicated that the incorporation of MDPB would not alter the bonding characteristics of the adhesives [103]. At the same time, handling characteristics of the adhesives were not hampered [100].

Based on this experimental adhesive system, the world’s first antibacterial adhesive system, whose primer containing 5% MDPB, was successfully commercialized (Clearfil Protect Bond, Kuraray Medical Inc.) in 2004 [100,101]. Clinical effectiveness of this commercialized adhesive system has been confirmed by many researchers [98].

Benzalkonium chloride (BAC) is a nitrogenous cationic surface-acting agent containing a quaternary ammonium group. It is often used as biocide, cationic surfactant and phase transfer agent [44]. BAC can inhibit MMPs in addition to their antibacterial effects [44,78]. Bisco (Schaumburg, IL, USA) is already manufacturing the commercial 37% phosphoric acid with 1 wt% BAC [44]. The etching agent with BAC may retain some of BAC’s MMP-inhibiting effect, possibly contributing to the longer durability of the hybrid layer [78].

Methacryloxylethylcetyl ammonium chloride (DMAE-CB) was incorporated in Single Bond 2 (3M ESPE, Minnesota, America) (3 wt%), showing long lasting antibacterial effect and no adverse effect on microtensile bond strength [104,105]. This adhesive exerted inhibitory effects on biofilm accumulation of *S. mutans* [97,104], The inhibitory mechanism might lay in the selective down-regulation of *gtf* gene expression of *S. mutans*, interrupting the synthesis of glucans [97]. The result of Live/Dead bacterial staining assay indicated that the DMAE-CB-incorporated adhesive exhibited detrimental effects on the growth, adherence and membrane integrity of *S. mutans* [98]. As the 50% toxic concentration for DMAE-CB against mouse fibroblasts is comparable to that of Bis-GMA [98], DMAE-CB would not compromise the biocompatibility of its parental adhesive [104]. Like other QAMs, its inhibition of MMPs at 0.5–5 wt% was illustrated [106].

The antimicrobial activity of quaternary ammonium compounds increases with the length of the alkyl moieties on the nitrogen atom [98]. Zhou et al. measured three-dimensional biofilms adherent on Scotchbond multi-purpose bonding agent SBMP) containing QAMs (10 wt%) with chain length (CL) from 3 to 18, identifying that increasing CL of QAMs greatly enhanced the antibacterial efficacy [54]. In the study of Li et al. there was the same trend [107]. The hydrophobicity-hydrophilicity balance, also referred to as “amphiphilic balance”, is a key factor in the antimicrobial activity and biocompatibility of polymeric QAS. The length of the substituted alkyl chain is one of the factors determining the hydrophobicity-hydrophilicity balance. Variation of the amphiphilic balance results in different affinity between the polycations and the bacterial membranes [98]. The longer hydrophobic alkyl chains facilitate polymeric QAS binding and diffusing through cellular membranes [54,98,107,108], and cause a higher surface charge density in the bonding agent [107]. MDPB monomers have alkyl chains of 12-carbon, which are longer than the two-carbon bridge of DMAE-CB, providing them with a better flexibility and adsorption of negatively-charged bacteria [98], whereas excessive hydrophobicity tends to block membrane penetration and increase cytotoxicity [108].

DMADDM with a chain length of 12 is a promising QAMs monomer. It has excellent antibacterial effects [52,54,55,107]. Many previous studies modified adhesive systems with DMADDM. The addition of 5 wt% DMADDM into adhesive Clearfil SE Bond (Kuraray Dental, Tokyo, Japan) had no adverse effect on adhesive bonding strength [109,110]. In addition, this antibacterial adhesive inhibited *S. mutans* biofilm growth, acid production and EPS synthesis through the whole development process of *S. mutans* biofilm [109].

The multispecies biofilm, which consist of *S. mutans*, *Streptococcus gordonii*, and *Streptococcus sanguinis*, can be inhibited by DMADDM containing SE Bond adhesive as well. Moreover, the ratio of caries associated bacteria *S. mutans* was decreased in multispecies biofilms [110]. DMADDM in adhesives could kill the early colonizing bacteria directly, and influence the development of biofilm indirectly by changing the charge density and surface roughness of the cured adhesives [109,110].

SBMP adhesive and primer containing 5 wt% DMADDM greatly inhibited dental plaque microcosm biofilm growth, metabolic activity, CFU and lactic acid production even with human salivary pellicle coverage [111].

The study into the cytotoxicity of DMADDM using human gingival fibroblasts revealed that DMADDM had much lower cytotoxicity than BisGMA [99,112].

Dimethylaminohexadecyl methacrylate (DMAHDM) with an alkyl chain length of 16 was mixed into SBMP adhesive and primer at 0 wt%, 2.5 wt%, 5 wt%, 7.5 wt%, and 10 wt%. The early-attachment coverage of bacteria greatly decreased with increasing DMAHDM mass fraction [113]. The new antibacterial primer and adhesive containing 10 wt% DMAHDM could reduce biofilm CFU by more than 4 orders of magnitude [113,114].

In comparison with other QAS monomethacrylates, quaternary ammonium dimethacrylate (QADM) has reactive groups on both ends of the molecule [115]. In addition, QADM has a low viscosity and is readily miscible with other dental dimethacrylates, thus improving processing, increasing range of filler loadings, and improving handling of the corresponding composite materials [95,115].

Cheng et al. integrated QADM into SBMP primer up to 10 wt%, and the dentin shear bond strength of adhesives did not decrease compared with the parent adhesive [56,95]. The uncured primer could kill the planktonic bacteria in caries cavities [95]. This result was further investigated by an in vitro study showing that the uncured primer containing QADM effectively reduce *S. mutans* impregnated into dentin blocks, compared to SBMP control primer [56]. The cured bonding agents containing QADM continued to have a strong antibacterial activity, thereby exerting a long-lasting effect against residual bacteria in the dentinal tubules as well as new invading bacteria along the margins due to microleakage [95].

#### 4.4.2. Chlorhexidine

CHX, a biguanide antimicrobial agent, has been broadly used in dentistry for its microbial efficacy and substantivity [116,117]. CHX is the most widely accepted non-specific MMP inhibitor even at a concentration of 0.05 wt% [36,38,41,64]. It can also inhibit cysteine cathepsins [31] and bacterial proteolytic enzymes [116]. The mechanism of chlorhexidine on MMPs inhibition is probably based upon the cationic-anionic reaction of CHX on glutamic acid residue of cysteine domain may deform MMPs molecules and prevent them from binding to substrates [66]. Another possible mechanism is that CHX could bind with calcium and zinc ions to MMPs, resulting in loss of catalytic activities of MMPs [118].

CHX not only can inhibit MMPs but also electrostatically binds to demineralized dentin [116,119]. This may be the reason for the long-term MMP inhibition efficacy of CHX in resin dentin bonds [41]. In some previous studies, CHX digluconate or CHX diacetate was added into etch-and-rinse adhesive systems (XP Bond and Ambar) [120] and the primer of two-step self-etching adhesives (Clearfil SE Bond) [32,121,122], in the etch-and-rinse adhesive systems the proper concentration of CHX digluconate is 0.2 wt% [120], but for the self-etch adhesives 0.1 wt% is just sufficient to preserve long-term dentin bond [122] without affecting the bond strength [32,120]. By this way, CHX would be entrapped in the polymer matrix, slowly leaching out from the polymer to the surroundings [120]. In addition, water-induced swelling may also lead to the release of CHX [120,123], along with the releasing of CHX, its MMPs inhibition effect may be weakened [123] and calcium ions released by the adhesive systems can also hamper CHX’s inhibition effect [121]. It has been demonstrated that CHX would influence the mechanical properties of bonding resin depending on its concentration [120]. CHX digluconate is only available as an aqueous solution which means mixing this solution with adhesives will increase water entrapment in the smear layer [120]. For CHX diacetate, it would increase water sorption of copolymers [124].

In addition to adding CHX into primer and bond, 2 wt% CHX has been incorporated into conventional phosphoric acid, after a two-year water aging, the result of microtensile bond strength and silver nitrate uptake testing indicate that the use of CHX in an acid conditioner was effective to reduce the degradation of dentin bonds [125].

#### 4.4.3. Doxycycline

Doxycycline (DOX), a tetracycline derivative, is widely used in the treatment of various infectious diseases. DOX could inhibit cariogenic bacteria such as *S. mutans*, *Lactobacillus acidophilus* and *Actinomyces viscosus* [35]. DOX is an inhibitor of MMPs by chelating zinc present in the catalytic domain of MMPs and the enzyme-associated calcium [35,68,69].

Feitosa et al. synthesized doxycycline (DOX)-encapsulated nanotubes and modified SBMP by incorporating the nanotubes into the adhesive resin dentin adhesive and demonstrated that this kind of adhesive could sustainably release DOX, thus inhibiting effect of MMPs [69] and the cariogenic bacteria.

#### 4.4.4. Hesperidin

HPN, a protein cross-linking agent previously mentioned, has been employed in modifying Clearfil SE primer [32]. In a pH cycling study, the lesion depth and mineral loss of bovine root dentin was evaluated by means of transverse microradiography (TMR). HPN showed remineralization of the surface and the subsurface lesion. It could be deduced that besides the effect of protein cross-linking, HPN has the capability of enhancing dentin lesion remineralization in vitro [73].

It was speculated that the effect of HPN on remineralization may be related with its interaction with collagen and/or noncollagenous proteins [73,74]. The stable dentinal organic matrix might hamper further diffusion of calcium and phosphate ions out of the dentinal lesion of caries, preventing further demineralization. Further, the preserved collagen matrix may be essential for the remineralization process, since it acts as a scaffold for mineral deposition [73,74].

#### 4.4.5. Zinc-Doped Adhesives

It is already demonstrated that the MMPs-mediated collagen degradation activity is dependent on calcium (Ca^2+^) and zinc (Zn^2+^) concentrations and the Zn/Ca ratio. Therefore, a relatively high concentration of Zn^2+^ may interfere with the MMP-mediated collagen degradation [71]. Zinc-doped adhesives can be obtained by using 20 wt% ZnO or 2 wt% ZnCl_2_ without altering adhesive physic, chemical and mechanical properties [126]. When using an etch-and-rinse adhesive procedure, zinc doping improves sealing efficacy and dentin remineralization. The slow Zn^2+^ liberation will facilitate the formation of a ZnO rich layer that permitting Ca and P deposits and further remineralization [127].

For self-etching adhesives, Zn should be added into the bonding resins, and never in the primer containing MDP, as it forms Zn-MPD complexes that may interfere with chemical interaction of calcium/dentine and MPD dentin infiltration [71,126].

#### 4.4.6. Combing Different Agents

It is difficult to find an agent with multiple desirable functions, so combining different agents to modify bonding systems may result in ideal effects to improve the durability of resin-dentin bond.

Cheng et al. have done a series of work to improve bonding systems with multiple different agents. They added quaternary ammonium dimethacrylate (QADM) and nanoparticles of silver (NAg) into the bond of SBMP [94]. Then, they combined QADM with NAg [56], DMADDM with NAg and NACP [52] to modify the primer of SBMP [56]. They also incorporated DMADDM and NAg into primer and bond of the same bonding agent [55], successfully enhancing the antibacterial potency of the bonding system, without adversely affecting the dentin bond strength [52,55,56,94]. Simultaneous utilization of different agents is promising to impart several constructive functions to bonding systems. According to previous studies about the functions of these agents, the primer with QADM and NAg [95] or DMADDM and NAg [55] may possess antibacterial properties and inhibit MMPs, at the same time, the primer containing DMADDM with NAg and NACP [52] probably can remineralize dentin lesions and inhibit MMPs.

Adhesive with double benefits of protein-repellent and antibacterial capabilities was achieved by use of protein-repellent 2-methacryloyloxyethyl phosphorylcholine (MPC) and antibacterial DMAHDM [128]. Furthermore, Wang et al. developed a novel rechargeable bonding agent with a combination of 5 wt% MPC, 5 wt% DMAHDM and 30 wt% NACP. Besides remineralization and acid-neutralization via NACP to inhibit caries, the multifunctional adhesive could reduce multispecies biofilm growth, metabolic activity, and polysaccharide production [129].

## 5. Conclusions

As composites restorations are the most popular materials to deal with caries nowadays, it is truly vital to understand the issues about the resin-dentin bond durability. The adhesive systems have developed for eight generations since early 1950s, dentin adhesive tend to simplify bonding steps, making them more user-friendly with long term bonding stability. However, degradation of resin-dentin bonding interface, microleakage and the negatively-influenced dental pulp are still the main reasons for failure of resin-dentin bonding. Therefore, large amount of studies devoted to modifying adhesive materials via following ways:

1. Incorporation of agents with anti-matrix metalloproteinase, remineralization, or antibacterial functions into adhesive systems. By this way, adhesives were exerted with particular function.

2. Adding multiple functional or combing different kinds of agents in adhesive systems are promising ways to providing more than one benefit functions and showing more effectiveness in improving the longevity of resinous restorations.

The currently laboratory data already holds excellent promise that stable resin-dentin bonds will be available in a near future. However, as the gap between laboratory tests and clinical results [130], more information should be gathered from in situ and clinical studies to investigate or commercialize the modified adhesives.

## Figures and Tables

**Table 1:** Development of dentin adhesives.

Generation of Adhesive System	Dates of Creation	Technique of Bonding	Bonding Mechanism
First	Early 1960s	No longer in use	Molecule interaction
Second	Early 1980s		Surface wetting phenomenon and ionic bond
Third	Late 1980s	Multiple steps selective-etch	Bonding to smear layer covered dentin
Fourth	Early 1990s	Three steps total-etch technique	Forming hybrid layer
Fifth	1990s	Two steps total-etch technique	Forming hybrid layer
Sixth	Start of 20s century	Two steps self-etching technique	Forming hybrid layer
Seventh	Start of 20s century	No-mix, one step self-etching technique	Forming hybrid layer
Eighth	Since 2010	Total-etch, selective enamel etching or self-etch technique	Forming hybrid layer
